# Hospital and Institutional News

**Published:** 1913-09-20

**Authors:** 


### HOSPITAL AND INSTITUTIONAL NEWS.

### YORK COUNTY HOSPITAL AND THE PUBLIC.

Occasion has been taken at the quarterly Court of the Governors of York County Hospital to make some reply to the local and general criticisms of inactivity which have been made against the house committee in regard to Sir Cooper Perry's Report. The resolution of the York Labour Party, referred to by us last week, which said that unless immediate steps were taken to carry out the Commissioner's suggestions they would advise the working-men to abstain from subscribing, was very fortunately timed, as it gave the quarterly Court a hint which it could not ignore. Mr. G. W. Lloyd, chairman of the sub-committee, which the house committee had appointed to consider the Report, repudiated the charges of inactivity. The committee, he said, had sat a great number of times, and in a very short time would issue a report to the house committee containing recommendations, some of which had already been carried out. Mr. H. Pratt asked when the report would be in the hands of the governors, and was told that it rested with the house committee to decide. The Lord Mayor, Sir J. Sykes Rymer, did not think any good came of trying to smother things up. He hoped as little delay as possible would be given to the issue of the report if one were to be issued at all. Mr. J. Bickle remarked that the public thought that something should be published, and that in the absence of anything the Workpeople's Hospital Fund had suffered. Dr. P. Macdonald, one of the honorary staff, also repudiated charges of inactivity, and affirmed that Sir Cooper Perry's "minor and unimportant recommendations" had been carried out, or were shortly to be so, and that he "was struck with the fine way" in which the hospital was administered.

### THE DEAN OF YORK'S STATEMENT.

We have given the above summary of the discussion because it reveals so clearly a resentment on the part of the speakers—except Mr. Pratt and, in some degree, the Lord Mayor—of the public demands for convincing proof of the committee's intention to prevent the possibility of such an inquiry being needed at a future time. The Dean of York, indeed, it is reported, saw fit to close the discussion with these remarkable words: "I cannot too deeply express my regret at the time taken up lately upon a question which has excited so many public minds." The reports of his speech go on to say that, apart from the reference to the mortuary incident, "with regard to all the rest of the Report it was mere trumpery, and they had spent £200 on it. It was an utter waste. All they had arrived at from the Report was that the diet wanted a little change." "There is nothing more to be said." We do not believe that the people of York will suffer such an attitude from the Chairman of the County Hospital without strong protest. It may be true that the Dean of York has arrived at nothing more from the Report than that the diet needs a little change. The public have arrived at a much more drastic opinion; and, in the face of this impenitent discussion, they will probably come back to *Truth's* opinion expressed not long ago, that only officers should be appointed to important posts in the institution who are in full possession of their faculties. What have the Workpeople's Hospital Fund and the York Labour Party to say? Such an attitude to public demands as was shown by this meeting, with the honourable exceptions referred to above, is nothing but an invitation to all classes of subscribers to cancel their subscriptions at once. The house committee's report was due last quarter; this quarter a public protest is met only by promises of further indefinite delay. The people of York must judge if this relation between a voluntary hospital and its supporters is to be permitted to endure.

### THE ESSEX HOUSE CASE AND ITS LESSON.

Dr. Henry Thomas Hamilton, the medical practitioner of Essex House, Barnes, who was indicted for being in charge of two lunatics within the meaning of the Lunacy Act of 1890, of unlawfully ill-treating them, and of taking charge for payment of a lunatic in an unlicensed house, and of committing an unlawful assault on one of them, has been acquitted. The jury stopped the case and found Dr. Hamilton "Not guilty." The verdict was received with popular demonstrations of applause. Evidence for the defence was given by Dr. Chambers, of Queen Anne Street, Cavendish Square, who had often visited Essex House as medical attendant to certified lunatics there, being appointed by the Lunacy Commissioners; by Mr. Hector Smith and Mr. C. W. Rowe, medical students at Charing Cross Hospital; Miss Levander, secretary and lady superintendent at Essex House; and several others. The announcement by the foreman of the jury, in answer to the Recorder's question: "We consider that the Crown have not made out their case," occasioned a scene of excitement which has had few if any parallels at the Central Criminal Court. Cheering and the waving of handkerchiefs lasted for several minutes, in spite of the shouts of the ushers, and at the first comparative silence the Recorder ordered the Court to be cleared, but the clearing was only the excuse for a further ovation which was paid to Dr. Hamilton on his appearance outside the Court. Medical students crowded round him, raised him on their shoulders, and shouting at the top of their voices carried him up and down the central hall. The case, we hope, may be of service in reminding all at the head of licensed houses of the public interest which is aroused in the well-being and kind treatment of the patients in this class of institution.

### A SET-BACK TO THE POOR-LAW MEDICAL SERVICE IN LONDON.

In June of this year the Camberwell Board of Guardians, with only two dissentients, decided to increase the salary of Dr. W. T. C. Keats, as medical superintendent of the infirmary, from £475 to £575. The Local Government Board have now written that they are not prepared to sanction this proposal in its entirety, but approve of the salary being increased to £525 per annum. This decision is greatly to be regretted. At a time when the need for increasing the efficiency of the Poor-Law Medical Service is becoming more generally realised and when the difficulty of attracting suitable candidates to it is increasing, it is of the highest importance that the Central Authority should stimulate the Guardians to offer higher salaries instead of acting as a deterrent influence. No one who is cognisant of the facts in this special case can have any doubt that Dr. Keats has well earned the increase that the Guardians wished to give him. He has now been in charge of the infirmary, which contains 828 beds, for about fifteen years and has had no increase in his salary for the last ten years. During the time he has held office he has revolutionised the work and administration of the infirmary, until to-day it is one of the best equipped and organised. It has been practically rebuilt, and the number of beds has been increased from 320 to the present total. The number of operations performed annually has risen from 120 to 500, the medical work has increased in the same proportion, and special departments have been added. In the provinces a higher salary than that suggested for Dr. Keats has been given. At Birmingham a medical superintendent was recently appointed at a salary of £600, rising to £850, with residential allowances. It is only in London that, owing to the operations of the Metropolitan Common Poor Fund, the Local Government Board constantly interfere with the discretion of the Guardians in the matter of officers' salaries. Unfortunately, this interference has practically always been in the direction of reducing the salaries the Guardians have thought it desirable to offer. This last instance of the obstacles placed in the way of those progressive London Guardians who are striving to increase the efficiency of their infirmaries cannot but have a disheartening effect upon the whole indoor medical service.

### THE PAYMENT OF BENEFITS TO HOSPITAL PATIENTS.

The approved societies have received from the Insurance Commissioners a notice to the effect that "on and after October 13, 1913, any sum withheld as sickness, disablement, or maternity benefit in the case of a person in a hospital or other institution, which is not expended in any manner authorised by Section 12 of the 1911 Act, must be paid in cash to the insured person after he leaves the institution, either in a lump sum or in instalments, as the society thinks fit."

### WEST HAM JURY'S UNDUE HASTE.

At a recent inquest held at West Ham Infirmary, Leytonstone, on a death in the infirmary the jury in a rider "thought West Ham Hospital should be censured for apparent lack of attention to a serious accident." It appears that immediately after a collision with a motor-cyclist, in which the deceased received certain injuries, he was taken to West Ham Hospital and transferred to West Ham Infirmary after being detained half an hour. The jury returned a verdict in accordance with the medical evidence that tuberculosis of the kidney was the cause of death. The accident probably accelerated the death, but it is a pity that more investigation was not made in this case preparatory to such a verdict.

### A CLERGYMAN'S VIEWS ON HOSPITAL SUNDAY.

The question of the decline of Hospital Sunday contributions has often been discussed in our columns, and the state of affairs in certain places where the congregational contributions from the churches are not in their right proportions can sometimes be strikingly shown. If the figures taken from a report issued by a certain church are to be regarded as a sample of the whole, the estimation in which hospitals are now held by congregations is no high one. We are told that £124 has been subscribed by a congregation for foreign missionary work, while for the local hospital, local dispensary, and Hospital Sunday Fund a total sum of £39 has been raised. Not the least noteworthy fact about these figures is that it was a clergyman who attracted our attention to this report. His comment may be given in his own words:—"The Church must fulfil her Lord's command to preach the Gospel to all nations, but she must not forget that same Lord's command to heal the sick."

### MINER'S PHTHISIS ON THE RAND.

That the tuberculosis problem is an economic one has been shown in a fresh way by the Rand labour troubles. Rumour says that many of the malcontents are men who have lost their health underground. The recently issued report of the Government Mining Engineer shows, however, that other than the actual workers have the matter at heart. As regards these last, it is the old story that Bacon summed up in the words "Great dangers demand great delights." The risk of invalidism necessitates large wages; the two together tempt to dissipation. We think that the risk is probably not adequately recognised by immigrants. All over the North of England, in the coal mining districts, consumptives may be met with or heard of who have come back from golden South Africa only to die. When they decided to go there they had no fear of underground work; that they had been familiar with from childhood. What they did not consider enough was the enormous difference between coal dust and the "stour" from rock-drills and rock-blasting—the one griming the face but soft and nearly innocuous to inhale; the other cutting the lung as emery would cut a sponge. The matter is, of course, one which should be left mainly in the hands of authorities like Mr. Kotze. His report seems to show a humanitarian and quite disinterested spirit. Medical co-operation would be useful in pointing his attacks upon the pure commercialist.

### A PIONEER OF THE MODERN HOSPITAL.

We regret to record the death of Dr. Frank Buszard, who had been for fifty years senior honorary physician to Northampton General Hospital, at the age of seventy-four. The son of a medical man, Dr. Buszard spent his whole life in active work in the hospital world, and was appointed house surgeon at Northampton General Hospital in 1862. The brilliance of his medical career may be judged by the fact that he was, in addition to an M.D. of London University and a Fellow of the Royal College of Surgeons, also a Fellow of the Royal College of Physicians. He studied at Guy's Hospital, and became qualified in 1861. We recorded in The Hospital during April 1912 the presentation of a portrait, from the brush of Mr. Harris Brown, to him by Lord Northampton. In reply to the speech which accompanied the presentation, Dr. Buszard emphasised the great importance of maintaining the voluntary system. How much he knew of this, and how intimate his experience was, can be gleaned from his recollections of fifty years ago, when the nurse was still nearly related to the charwoman, and when the night nurse was generally a person who added to her income by working "overtime" in the night hours. A prominent speaker at medical congresses, Dr. Buszard will be remembered as a pioneer of the modern hospital, and with him one of the few remaining men of his generation in the hospital world has passed away.

### A SCOTCH HOSPITAL BENEFACTOR.

With the death of Mr. Peter Coats a fine example of the type of enthusiastic worker produced by the voluntary hospital system passes away. Following his father, the late Sir Peter Coats, he was a most munificent benefactor of the Royal Alexandra Infirmary, Paisley, and he might almost be said to have built the institution. The isolation block, the mortuary chapel, indeed the whole pathological laboratory, were erected through his generosity, and the nurses' home, which is one of the infirmary's most prominent and costly features, was also built by him. Though it is, of course, with his hospital benefactions that we are here concerned, it may be added that the town also is his debtor for many buildings, and there was perhaps no form of activity which would appeal to him in vain. He was seventy-two at the time of his death.

### THE COST PER BED IN LONDON HOSPITALS.

The latest statistical report of King Edward's Hospital Fund contains figures which deserve being put upon record, whatever may be the deductions from them, with which, at the moment, we are not concerned. The increase in the cost per bed occupied, which was noticed first two years ago, and per attendance of out-patients, has continued, and represents for the 109 London hospitals considered by the report a total of some £21,800. The greatest average increases in the cost per bed appears in the accounts of the different types of institutions under different sub-heads, which may be summarised as follows. The larger general hospitals show the greatest average increase under provisions; hospitals for women under surgery, dispensary and salaries; cottage hospitals under provisions and salaries. The greatest increases in the cost per out-patient attendance appear, in the case of large general, chest, and lying-in hospitals, under salaries; and in the case of children's hospitals under surgery and dispensary. The appended table gives in detail the increases in cost as compared with 1911:—

| | Average cost per bed | | | | | | Average cost of out-patient attendance | |
|---|---|---|---|---|---|---|---|---|
| | 1912 | | | | | | 1912 | |
| | £ | s. | d. | £ | s. | d. | Pence | Pence |
| Larger General | 82 | 1 | 4 | +2 | 15 | 2 | 7·43 | +·32 |
| Smaller General | 74 | 6 | 11 | +0 | 17 | 2 | 5·54 | +·13 |
| Consumption | 60 | 16 | 11 | +1 | 10 | 8 | 11·79 | +·67 |
| Hospitals for Women | 77 | 13 | 0 | +2 | 9 | 8 | 7·91 | +·26 |
| Children's | 67 | 13 | 0 | −0 | 17 | 1 | 7·[illegible]6 | +·28 |
| Ophthalmic | 56 | 10 | 11 | −1 | 4 | 0 | 5·45 | +·22 |
| Hospitals for Epilepsy | 70 | 13 | 3 | +0 | 1 | 1 | 8·29 | +·13 |
| Cottage | 66 | 19 | 10 | +3 | 14 | 8 | 3·80 | +·42 |
| Lying-in | 64 | 13 | 7 | −1 | 4 | 2 | 9·35 | +·53 |

The lessons to be drawn from the report and these figures we shall discuss in a later issue.

### THE LATEST RECRUIT TO THE UNIFORM SYSTEM.

The committee of management of Ancoats Hospital, Manchester, have unanimously decided to adopt the Uniform System of Hospital Accounts. Moreover, we understand that in order to come quite into line with other institutions for comparison purposes, they have resolved to close their financial year on December 31 in future, instead of June 24, as hitherto.

### HOSPITALS AND MILK-CONTRACTS.

Somewhat unexpectedly St. Bartholomew's Hospital has become the temporary centre round which discussion and criticism as how best to secure a pure milk supply for institutions and the general community are concentrated. No sooner were the precautions taken by St. Bartholomew's Hospital to secure a clean milk supply published than the value of these precautions was challenged, mainly on the ground that "bacteriological examination of the milk is the only method of checking its real cleanliness." Thus the nature of the contract is seen to be the most important thing. On this technical and involved subject, about which it must be remembered expert opinion is not altogether agreed, readers of The Hospital have the valuable advantage of having recently had placed before them a discussion of the whole principles and practice of "Milk-Contracts" in the very well-informed and suggestive series of articles which Dr. Everitt E. Norton, tuberculosis county medical officer for Middlesex, recently published in our columns. His detailed criticisms need not be repeated here, but it will be remembered that he frankly faced the economic difficulty, which is the rock on which so many attempted reforms have broken down. The price of milk in fact is tending to rise in any case, as, for instance, in Leicestershire at this moment, where an increase of from 3d. to 4d. a quart has been decided on, due, it appears, to the number of cows which have been condemned under the Tuberculosis

Order, for which the owners say they will get no compensation. The moral, in short, is that pure milk is an expensive and rare article, and as such will go up in price, much as new-laid eggs have done, and probably will continue to do.

### THE PUNISHMENT OF WORKHOUSE INMATES.

The regulations for the maintenance of discipline in the workhouse in the Draft Poor-Law Institutions Order reproduce almost exactly those contained in the General Consolidated Order. However, playing at cards or other game of chance by an inmate is no longer enumerated amongst the list of offences constituting disorderly conduct. An alteration of some importance has been made in the section requiring, in certain defined cases, the certificate of the medical officer that punishment is not likely to lead to injury to health before its infliction. In the General Consolidated Order this certificate is required in the case of any woman "who may be pronounced by the medical officer to be pregnant." In the Draft Order the words "an inmate who is advanced in pregnancy" is substituted for this phrase. No definition of "advanced in pregnancy" is given, and it seems as if it would be left to the workhouse master to place what construction he likes upon the phrase. In this case the wording of the General Consolidated Order is obviously preferable. On the other hand, the Draft Order improves upon the Consolidated Order by including "women recently confined" amongst the classes for whom a medical certificate is required before punishment can be inflicted, but it would be better if a definite period of time were mentioned.

### THE SURGICAL PROBLEM IN RURAL WORKHOUSES.

The difficulty of providing properly for the sick in the small rural workhouses is well illustrated by recent events in the Mutford and Lothingland Union in Suffolk. A short time back the Guardians decided that no operations were to be performed in the workhouse, but that cases requiring operation were to be removed to the local hospital. Against this decision the workhouse medical officer wrote in protest that if no operations were performed in the workhouse the nurses would be deprived of much that gave interest to their work and the discontent amongst them would be increased. He added, however, that during the last five and a half years he has had to perform only twenty or twenty-five major operations. We can sympathise with the medical officer in his desire to retain these surgical cases, but this admission cuts away the grounds of his objection to their removal. Discontent amongst the nursing staff owing to lack of surgical work can hardly be mitigated to any appreciable extent by four or five major operations a year. A more important point is that the Guardians cannot be expected to provide instruments and the appliances necessary for aseptic surgery if surgical cases are so few in number. Under the circumstances the Guardians' proposal seems reasonable. The chief objection to it is that even in small workhouses there must occasionally arise in the sick wards surgical emergencies in which the removal of the patient must be attended with risk. The Guardians' proposal may be the best possible under present conditions, but the solution really lies in the amalgamation of Unions over a sufficiently large area to permit of the provision of an infirmary properly equipped and staffed and able to treat efficiently the sick poor from all the amalgamated Unions.

### THE MEDICAL OFFICER AS STOCKTAKER.

The Braintree Guardians recently requested their medical officer to take stock of the drugs at the workhouse. This he at once declined to do, and the Guardians appealed to the Local Government Board, which has forwarded a reply to the effect that the Guardians might require the medical officer to state the quantity of drugs he has in stock. This decision is to be regretted. A medical man is capable, of course, of performing the duties of a stocktaker, just as he could those of a gate porter if required, but his time might be better employed in the skilled work for which he has been trained. A medical officer's time is usually very fully occupied, and the hours spent in estimating and recording the stock of drugs, which could easily be done by any intelligent member of the clerical staff, means so much the less time available for work in the wards.

### HOW TO SPECIALISE IN TUBERCULOSIS.

What is described as a course of lectures "especially for candidates for tuberculosis officerships" has been arranged by the Royal Institute of Public Health. There are announced to be eleven lectures, two of them by the tuberculosis officers of Kent, the fee for the course being three guineas. Professor Sims Woodhead's is the first name. At its close there will be an optional examination, to those successful at which certificates will be granted. This arrangement does not seem likely to lead to any good. To begin with, the lecture is out of date; nowadays text-books have supplanted it altogether. The subjects are far too large to be covered in an hour's talk, even by a Chrysostom; and this makes it matter the less that one stumbling-block to the incompetent tuberculosis officer—namely, laryngology—escapes mention. The proper way for a medical man to specialise in tuberculosis is to begin with a junior post at sanatorium, chest hospital, or dispensary; to get as much clinical and laboratory experience as ever he can, and to keep up diligently meanwhile with the chief current literature, home and foreign, of his subject, to this end subscribing to a French or German special journal, it being, of course, premised that he has already grounded himself by study of one of the special text-books, such as Cornet's or Bandelier and Roepke's work, or one of the American handbooks. Compared with this, all short courses of instruction (and similarly, therefore, with their certificates) are of no value. An *exequatur* awarded by an educational body of standing, after a real examination, practical and theoretical, and taking into count (this, however, is too much to hope for)

original as well as set work, would be another matter. But the time for adding another diploma to those of tropical medicine and of psychiatry is not yet. Perhaps oto-rhino-laryngology will come next. Certain tuberculosis workers are, however, looking forward to something of the kind as a useful regularising measure. There is something about the subject of tuberculosis—some magnetism, some mirage of ready capture and facile discovery, some demoralising influence, some *Erregung*—which sets medical men talking nonsense (a fault generally confined to laymen) as no other subject does.

### ONE OF THE EVILS OF BAD COOKING.

A medical officer of health for one of the London districts in speaking of infant mortality lately said there was a large decrease in 1912 as compared with the previous year. He thought that Nature had mercifully interfered to save many young lives from ignorant mothers by sending a wet and cool season during the summer months. He went on to speak of the misery and muddle of many a working-man's home due to the profound ignorance of the wife on the most elementary knowledge of household management. How is it to be expected that girls who leave school and go straight to factories and workshops without being taught the duties of wifehood and motherhood can possibly make fitting wives for the working-men? They marry from the factory often without any knowledge whatsoever of the plainest cooking. The man's lot is a most miserable one, even where, in many cases, his wages entitle him to a comfortable home. It is quite probable that a large amount of drunkenness is due to bad cooking. What do our readers think?

### MEASLES AND SCHOOL CLOSURE.

Among the now considerable number of eminently practical publications issued by the Medical Officers of Schools Association, the latest addition, on the control of measles, must be considered as one of the most interesting, not only to school doctors, but also to teachers and parents. This disease stands in a peculiar position in regard to public health endeavour, for it is agreed by authorities that it is a practical impossibility to stamp out the disease. But more than this, it would be nothing less than dangerous to attempt to do so, for even if it were possible to secure exemption for a time, say for a generation or two, the eventual and inevitable introduction of the infection from without, to say nothing of a possible re-evolution of the specific poison, would result in a catastrophe not less lamentable than that which followed its implantation on a virgin soil, such as that afforded by the unfortunate inhabitants of Fiji. Although the discussion in the pamphlet now published by J. and A. Churchill, 1s., shows considerable divergence of opinion on matters of detail, we may take one of the papers—that by Dr. C. J. Thomas—as representing the soundest views on the subject of the control of measles. The papers are well worth reading, for this subject is one which is of the greatest importance to a very large section of the community, and a mere statement of conclusions is difficult of appreciation without the simultaneous comprehension of the arguments and ascertained facts. One thing, however, stands out clearly and ought to be insisted upon, and that is the futility, nay, disadvantage, of school closure when measles is prevalent. It is time that school managers should realise, as has been proved over and over again, that very little can be done in urban areas to arrest the spread of measles or to reduce the attack rate, but, on the other hand, that much can be done if knowledge of the cases is available, through health visitors, etc., in the way of reducing the fatality of measles, and that therefore it is in every way desirable to continue the organisation which is the only source of that knowledge, namely, the machinery relating to school attendance. So many superstitions still exist in regard to measles, that nothing but good can come of the dissemination of a little more light on the problems of its incidence, age of attack, and manner of spread.

### THREATENED STRIKE OF MENTAL HOSPITAL WORKERS.

A strike among mental hospital workers is threatened in a letter which Mr. George Gibson, the organising secretary of the National Asylum Workers' Union, has issued to the Yorkshire papers. He states that the mental hospital workers in the West Riding of Yorkshire have unanimously decided to refuse the " alleged concessions " which have been proposed by the Asylums Board to allay the prevalent discontent. The object of his letter Mr. Gibson states to be a warning to the public of the gravity of a situation which knowledge in his possession that the staffs have decided to enforce their demands by drastic steps leads him to disclose publicly. He adds that a special meeting of the executive council of the Union has been called for September 27, when " the demands of the West Riding members for a strike " will be considered. He further adds that the executive council " will have no other course open to them than to sanction the demand "—a statement that appears intended to close the door to further discussion. It should be remembered that the National Asylum Workers' Union is a youthful trade-union organisation, which has already been heard of at Cardiff, and which was founded in 1911. It must not be confounded with the Asylum Workers' Association, which has been in existence for practically twenty years. To mental hospital workers generally the policy threatened by Mr. Gibson will be regarded as irresponsible in aim and presentation, and we still have hopes that wiser counsels may prevail to the lasting settlement of legitimate grievances.

### THE BRITISH ASSOCIATION AT BIRMINGHAM.

It is in no unfriendly spirit that we relegate to a Note the proceedings of the British Association at Birmingham. The Association has for its function the popularising of scientific studies in the numerous branches of research with which it deals, and consequently its discussions are of more interest to the lay than to the professional or institutional public. The discussions, moreover, labour under the defects of their usefulness. It is inevitable, as we pointed out last year, that there should be a temptation to play to the gallery, a temptation to which the columns and headlines of the daily newspapers have borne abundant witness during the past week as usual. The Association, in short, is more concerned with the elementary education of the public than, as was the case with the recent International Congress of Medicine, with the higher education of professional men. This function is, of course, an extremely useful and proper one, but it means to the hospital world that there is very little, if anything, new to be learnt from its discussions. Such subjects as "the origin of life" continue to exert their fascination upon the lay mind, and again this year we have it debated by Professor B. Moore, and to hear his address the three Sections of Zoology, Physiology, and Botany were assembled. The Piltdown skull was also discussed by Dr. Smith Woodward, and these provide two good instances of the nature of the discussions encouraged by the Association. The conclusion of the whole matter may perhaps be summed up as this. In different branches of science we are able to extend our knowledge, with the result that the limits of the unknown are thrust gradually further back; but when we try to co-ordinate these extensions of knowledge so as to arrive at a closer understanding of in what life itself consists, we seem no nearer than we ever were to its solution. It is, however, the solution which excites the public, and this is why the temptation always exists to pretend to a greater knowledge than is really ours, or likely to be ours for an indefinite period.

### PROPOSED MILK FARM AT CARDIFF.

MR. A. J. ATWELL has been asked to present some data with reference to his suggestion that King Edward VII.'s Hospital, Cardiff, should acquire a farm for the purpose of providing milk for the institution. At last week's meeting of the board of management Mr. Atwell remarked that about four hundred gallons of milk per week had to be provided for the hospital, which at one shilling per gallon mounted up to a very large sum in the course of the year. He added that he understood, moreover, that a very good profit went to the farmer who supplied the milk. He suggested that the house committee should appoint a sub-committee to consider the matter, as he thought such a farm could provide, not only milk, but fruit and vegetables much cheaper than it was possible to purchase them in the market. In answer to General Lee's question: "Where do you propose to get the money?" Mr. Atwell replied that he thought local philanthropists would provide the money. It was then announced that the house committee had the matter in hand. The late Samuel Butler used to say that he knew of no exception to the rule that it was cheaper to buy milk than to keep a cow. But conditions change, and now that hospitals are being forced to recognise that really strict conditions of cleanliness, if carried out, are bound to raise the price of milk considerably, it is perhaps time to reopen a question which many have considered, like Samuel Butler, to be closed. As to co-operative farming, that is again another matter, as one of the speakers pointed out, and it was one which was dealt with in a special series of articles which appeared in THE HOSPITAL a few months ago. We therefore venture to direct Mr. Atwell's and the committee's attention to an article in our issue of January 4 last, entitled "Do Hospital Dairies Pay?" and to its successor in the same series, on "Hospital Gardening and the Supply of Vegetables," which was published in the issue of January 18. Faced with the problem their views would be welcomed by other hospitals, and we invite their expression in our columns.

### MENTAL HOSPITAL SUPERINTENDENT RETIRES.

DR. GEORGE EDWARD CARRE, medical superintendent of the Tyrone and Fermanagh Mental Hospital, has sent in his resignation, to take effect from November 15 next. Dr. Carre, who has seen thirty-three years' service in Omagh, held previous institutional appointments as visiting surgeon to the Letterkenny Mental Hospital, and at the Castlebar Mental Hospital, where he was for six years. A resolution expressing the committee's sincere regret was passed, but they felt that he had "earned a rest," as all will feel who appreciate the strain of mental work. Dr. Carre graduated M.B. at Dublin in 1858, and has spent practically all his life in Ireland.

### MEDICAL OFFICER AS M.P.

IT is announced that Mr. Burnet, the medical officer of health for Tunbridge Wells, to which he came eighteen months ago, has been selected as Liberal candidate for Warwick and Leamington at the next general election. His medical education took place at Edinburgh, and after holding the post of resident surgeon at the Maternity Hospital there, and having taken a degree in Public Health, he devoted himself largely to school and public health work. As we have always maintained that the personnel and the debates of the House of Commons would be much improved by the presence of more medical members of Parliament, we are able, quite apart from party, to welcome Mr. Burnet's candidature.

### THIS WEEK'S DRUG MARKET.

BUSINESS in the Drug market has been rather better during the week, but the fluctuations in prices have not been of an important character. The values of cream of tartar and citric and tartaric acids are firmly maintained, and any change in the immediate future would no doubt be in an upward direction. Extreme prices are quoted for Russian cantharides as a result of the smallness of the supply. Quillaia bark is scarce and dear. Oil of caraway is slightly dearer, but oil of cloves is rather lower in price. Business has been done in ipecacuanha at good prices. Menthol seems to be tending downwards in price. High prices are quoted for orris root. The market for cod-liver oil is extremely quiet, but there is practically no change in price.

